# Psychological long-term effects of a no-sedation protocol in critically ill patients

**DOI:** 10.1186/cc9779

**Published:** 2011-03-11

**Authors:** T Strøm, M Stylsvig, P Toft

**Affiliations:** 1 Odense University Hospital, University of Southern Denmark, Odense, Denmark

## Introduction

A protocol of no sedation has been shown to reduce the time patients receive mechanical ventilation and reduce intensive care and total hospital length of stay [1]. The long-term psychological effect of this strategy has not yet been described.

## Methods

We contacted all surviving patients who had been randomized to our original trial that compared a no-sedation strategy with a traditional strategy of sedation and daily wake-up trial. Patients were offered a follow-up interview with a neuropsychologist. The neuropsychologist was blinded to the randomized treatment. All patients were assessed with the same validated psychological tests. Post-traumatic stress disorder (PTSD) was evaluated with three tests: Revised Impact of Event Scale, State Anxiety Inventory Scale and Post-Traumatic Stress Syndrome 10-Questions Inventory scale (PTSS-10). The generic quality of life was evaluated using the Medical Outcomes Study 36-item short-form health survey (SF-36). Depression was evaluated using the Beck Depression Inventory-2 score (BDI-II). Patients were also assessed with a modified version ICU memory tool.

## Results

A total of 26 patients were interviewed (13 from each group). The time span between randomization and interview was 2 years (no-sedation group 1.78 (1.46 to 2.10) years vs. sedated group 2.04 (1.55 to 2.29) years, *P *= 0.32). No difference was found with respect to baseline data. Very few patients suffered from PTSD and no significant difference was found between the two groups. No difference was found with respect to generic quality of life (SF-36). A very low rate of depression was found in both groups with no significant difference. The modified ICU memory tool showed that two-thirds of patients from both groups had experienced nightmares during their ICU stay. Very few patients remembered pain or breathing difficulties in the ICU (NS).

## Conclusions

Our data disprove the popular supposition that a protocol of no sedation applied to critically ill patients undergoing mechanical ventilation increases the risk of long-term psychological sequelae after intensive care compared to standard treatment with sedation. With the reduced ventilator days, reduced ICU and hospital length of stay, this psychological follow up further supports the benefits from a no-sedation strategy applied to critically ill patients undergoing mechanical ventilation.
